# Building a risk prediction model for anastomotic leakage postoperative low rectal cancer based on Lasso-Logistic regression

**DOI:** 10.1186/s12876-025-04128-y

**Published:** 2025-07-30

**Authors:** Zhenhao Quan, Lin Lin, Renwei Huang, Kaiyu Sun, Feipeng Xu

**Affiliations:** Department of Gastrointestinal Surgery, Affiliated Hospital of Guangdong Medical University, No. 57 South Renmin Avenue, Xiashan District, Zhanjiang, Guangdong Province 524001 China

**Keywords:** Low rectal cancer, Anastomotic leakage, Nomogram

## Abstract

**Objective:**

To build a nomogram model for predicting the risk of anastomotic leakage (AL) postoperative low rectal cancer based on Lasso-Logistic regression.

**Methods:**

A total of 482 patients with rectal cancer who underwent low rectal cancer surgery in our hospital from June 2017 to May 2023 were selected as the training set, and 127 patients with rectal cancer who underwent low rectal cancer surgery in our hospital from June 2023 to April 2025 were selected as the validation set. According to whether AL occurred postoperative, the patients in the training set were divided into AL group (*n* = 54) and N-AL group (*n* = 428). The data of each group were collected, and the influencing factors of AL in patients postoperative with rectal cancer in the training set were analyzed by Lasso-Logistic regression model. H-L goodness-of-fit test, ROC curve and calibration curve were used to analyze the discrimination and consistency of the model. The nomogram model was validated using the validation set. The DCA curve was used to evaluate the clinical utility of the model.

**Results:**

In the training set, the AL group had a higher proportion of patients with tumor stage ≥ T3 and longer operation times compared to the N-AL group; additionally, fewer AL patients had a protective stoma, and the tumor was located a shorter distance from the tumor to the anal verge than in the N-AL group. (*P* < 0.05). Lasso-Logistic regression analysis showed that when the penalty coefficient λ = 0.02735463, the model demonstrated good performance, gender (*OR* = 3.107), NRS2002 score (*OR* = 8.619), protective stoma (*OR* = 0.297), distance from tumor to anal verge (*OR* = 0.284), operation time (*OR* = 1.033) were the influencing factors of postoperative AL in low rectal cancer (*P* < 0.05). The 5 influencing factors were introduced into R software to establish a nomogram model for the risk of postoperative AL in low rectal cancer. The area under the ROC curve was 0.940. H-L goodness of fit test showed that there was no significant difference between the predicted value of the model and the actual observed value (*χ*^*2*^ = 6.438, *P* = 0.598). The slope of the calibration curve was close to 1. The validation set showed that the nomogram model had good discrimination and consistency. The DCA curve showed that the model had high clinical utility and net benefit when the risk threshold was between 0.08 and 0.85.

**Conclusion:**

Gender, NRS2002 rating, diverting ostomy, distance from tumor to anal margin, and operation time are all influencing factors of postoperative AL in low rectal cancer. The nomogram prediction model based on Lasso-Logistic regression has high consistency, discrimination and clinical application value.

**Supplementary Information:**

The online version contains supplementary material available at 10.1186/s12876-025-04128-y.

## Introduction

Data indicate that rectal cancer ranks among the leading cancers in terms of incidence rates in China, with high mortality rates and poor prognosis [1]. In recent years, with the iterative development of surgical concepts and techniques, surgical success rates have steadily improved, and the rate of sphincter preservation has increased. This advancement means that some patients no longer need to undergo permanent ostomy, resulting in less impact on their postoperative quality of life. However, certain surgeries, such as laparoscopic procedures, still cannot entirely prevent the occurrence of anastomotic leakage (AL) after surgery, particularly in cases of low rectal cancer [2]. At the onset, AL presents with diverse clinical manifestations, ranging from occult leakage below a functioning stoma to fecal peritonitis accompanied by multiple organ failure. Silent leakage in the initial stages may progress to chronic pelvic sepsis. Consequently, AL is a severe complication, with an incidence rate of 1–30% [3]. Studies have shown that AL prolongs postoperative hospital stays, delays the timing of adjuvant chemotherapy and radiotherapy, adversely affects postoperative quality of life, and significantly increases the rates of local recurrence and mortality [4, 5]. Given these adverse effects associated with AL, reducing its postoperative incidence has become a critical concern for clinicians [6]. A key to reducing the incidence of postoperative anastomotic leakage (AL) is to identify its influencing factors and assess the associated risk. Greijdanus et al. [7] evaluated individual survival risk in AL patients using a stoma scoring system. However, many previous studies have focused on isolated risk factors for AL and were conducted years ago, limiting their applicability. With advances in medical technology, particularly the widespread adoption of laparoscopic surgery, the risk factors associated with AL have been evolving over time. Therefore, earlier studies have limited predictive value for current AL risk assessment. A nomogram is a medical tool that converts data into a graphical format, allowing the incorporation of multiple influencing factors. It is commonly used to predict various postoperative complications. This study includes data from patients with and without AL following surgery for low rectal cancer. It analyzes the preoperative, intraoperative, and postoperative differences between these groups, aiming to provide a theoretical basis for reducing the risk of AL in low rectal cancer patients.

## Materials and methods

### General information

**Fig. 1:**
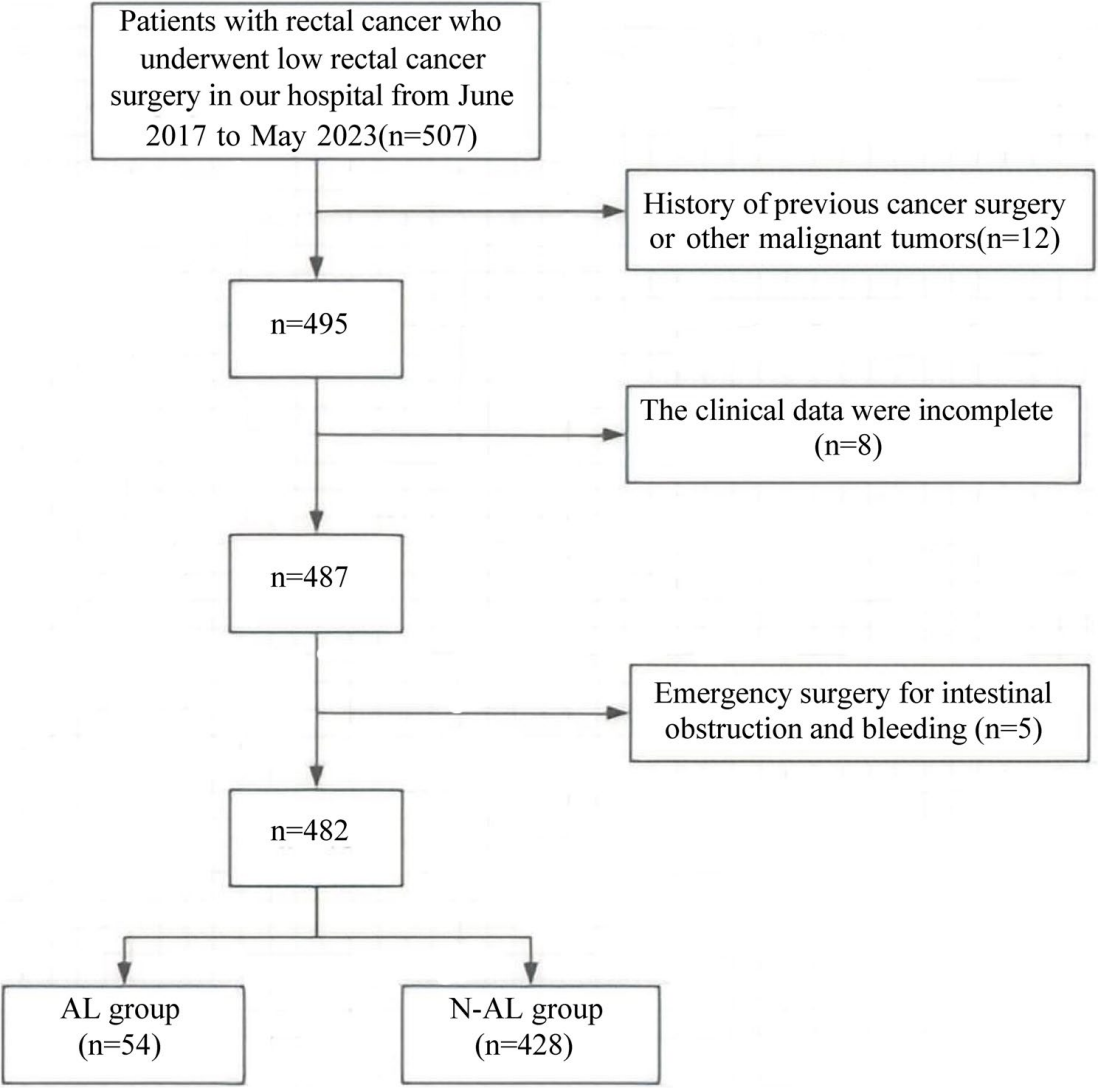
Case screening process

A total of 482 patients with low rectal cancer who underwent surgical treatment at our hospital between June 2017 and May 2023 were selected as the training cohort. All patients met the inclusion and exclusion criteria. Based on the occurrence of anastomotic leakage (AL) within 30 days postoperatively, patients were divided into an AL group (*n* = 54) and a non-AL (N-AL) group (*n* = 428). Clinical data were collected from both groups to develop a nomogram model. The inclusion criteria were as follows: (1) postoperative pathology confirmed rectal cancer; (2) patients with M0 stage; (3) tumor located ≤ 8 cm from the anal verge; (4) underwent laparoscopic low anterior resection; (5) aged between 18 and 80 years; (6) complete medical records available; (7) AL cases met the diagnostic criteria for AL [8]: These patients presented with local pain, abnormal drainage, systemic fever or signs of infection, and gastrointestinal dysfunction, and were diagnosed with Grade B or C anastomotic leakage based on CT scan findings; (8) Patients who underwent surgical treatment performed by the same team of surgeons at this hospital; (9) Intraoperative fluorescence imaging was performed in all cases. Exclusion criteria included: (1) history of prior cancer surgery; (2) emergency surgeries for conditions such as bowel obstruction or bleeding; (3) combined organ resection; (4) other malignancies; (5) distant metastases; (6) Underwent transanal total mesorectal excision (TME) technique. Additionally, a separate cohort of 127 patients who received surgical treatment for low rectal cancer at our hospital between June 2023 and April 2025 was used as a validation set to evaluate the performance of the model. The study was approved by the ethics committee. The case screening process is shown in Fig. 1.

### Methods

Data were collected from the hospital case management system, including: (1) Demographic and clinical information: age, gender, BMI; (2) Lifestyle and comorbidities: smoking, alcohol consumption, hypertension, diabetes; (3) Tumor and surgical characteristics: American Society of Anesthesiologists (ASA) classification [9] (low risk: grades 1–2; high risk: grades 3–4), maximum tumor diameter, pathological type, clinical staging (tumor T stage and tumor N stage), vascular invasion, nerve invasion, preoperative albumin, preoperative hemoglobin, nutritional risk as assessed by the Nutritional Risk Screening 2002 (NRS2002) [10] (a score of ≥ 3 indicates nutritional risk), and preoperative nutritional support; (4) Neoadjuvant therapy (including chemotherapy alone and chemoradiotherapy), protective stoma, tumor distance from the anal verge, operative time, intraoperative blood loss (based on clinical practice: intraoperative blood loss within 100 mL is generally considered relatively safe for most patients, while blood loss exceeding 100 mL may increase the risk of complications; therefore, 100 mL was used as the threshold in this study, and patients were categorized as < 100 mL or ≥ 100 mL), presence or absence of intestinal obstruction, and history of prior rectal resections. (5) Anastomosis methods, including the Double-Stapling Technique (DST) and hand-sewn Parks procedure.

### Statistical analysis

Data were processed using SPSS 22.0. Continuous variables were expressed as mean ± standard deviation ($\overline x\pm S$) and analyzed using t-tests. Categorical data were expressed as n (%) and analyzed using chi-square (χ²) tests.LASSO-logistic regression was used to select the influencing factors. Prediction models were constructed using R software (version 4.5.0) with the rms package. Model discrimination, calibration, and clinical utility were assessed using the receiver operating characteristic (ROC) curve, calibration curve, and decision curve analysis (DCA), respectively. A significance level of *P* < 0.05 was considered statistically significant.

## Results

### Baseline clinical characteristics of the AL and N-AL groups in the training set

In the AL group, there was a higher proportion of male patients, patients with a tumor maximum diameter ≥ 5 cm, patients with tumor stage ≥ T3, and patients with an NRS2002 score ≥ 3; additionally, the operative time was longer compared to the N-AL group. In contrast, the tumor was located a shorter distance from the anal verge, and fewer AL patients had a protective stoma (*P* < 0.05). For details, see Table 1.

**Table 1:** Baseline clinical characteristics of the AL and N-AL groups in the training set

Factors		AL group(*n* = 54)	N-AL group(*n* = 428)	χ^2^(t)	*P*
Age					
< 60	272	32(59.26)	240(56.07)	0.198	0.657
≥ 60	210	22(40.74)	188(43.93)		
Gender					
Male	296	42(77.78)	254(59.35)	6.874	0.009
Female	186	12(22.22)	174(40.65)		
BMI(kg/m^2^)		23.24 ± 3.12	23.16 ± 3.28	(0.170)	0.865
Smoking					
Yes	111	17(31.48)	94(21.96)	2.451	0.117
No	371	37(68.52)	334(78.04)		
Tipple					
Yes	79	11(20.37)	68(15.89)	0.703	0.402
No	403	43(79.63)	360(84.11)		
Hypertension					
Yes	131	16(29.63)	115(26.87)	0.185	0.667
No	351	38(70.37)	313(73.13)		
Diabetes					
Yes	92	12(22.22)	80(18.69)	0.387	0.534
No	390	42(77.78)	348(81.31)		
ASA classification					
1 ~ 2	405	30(55.56)	275(64.25)	1.561	0.212
3 ~ 4	177	24(44.44)	153(35.75)		
Maximum tumor diameter					
< 5 cm	358	31(57.41)	327(76.40)	9.054	0.003
≥ 5 cm	124	23(42.59)	101(23.60)		
Pathological type					
Adenocarcinoma	399	42(77.78)	357(83.41)	1.068	0.302
Mucinous adenocarcinoma	83	12(22.22)	71(16.59)		
Tumor T stage					
≤T2	188	14(25.93)	174(40.65)	4.372	0.037
≥T3	294	40(74.07)	254(59.35)		
Tumor N stage					
N0	182	22(40.74)	160(37.38)	0.230	0.632
≥N1	300	32(59.26)	268(62.62)		
Vascular infiltration					
Yes	116	15(27.78)	101(23.60)	0.458	0.498
No	366	39(72.22)	327(76.40)		
Neuroaggression					
Yes	118	15(27.78)	103(24.07)	0.357	0.550
No	364	39(72.22)	325(75.93)		
Preoperative albumin(g/L)		39.21 ± 4.32	39.73 ± 4.48	0.807	0.420
Preoperative hemoglobin(g/L)		125.69 ± 11.14	127.21 ± 12.24	(0.868)	0.386
NRS2002 rating					
< 3	261	9(16.67)	252(58.88)	34.413	< 0.001
≥ 3	221	45(83.33)	176(41.12)		
Preoperative nutritional support					
Yes	66	7(12.96)	59(13.79)	0.027	0.868
No	416	47(87.04)	369(86.21)		
Neoadjuvant therapy					
Yes	63	9(16.67)	54(12.62)	0.692	0.405
No	419	45(83.33)	374(87.38)		
Prophylactic stomy					
Yes	421	38(70.37)	383(89.49)	15.851	< 0.001
No	91	16(29.63)	45(10.51)		
Distance from tumor to anal margin(cm)		3.91 ± 0.87	5.26 ± 1.23	(7.819)	< 0.001
Operation time(min)		179.87 ± 43.38	138.26 ± 37.21	(7.594)	< 0.001
Intraoperative bleeding(mL)					
< 100	94	7(12.96)	87(20.33)	1.656	0.198
≥ 100	388	47(87.04)	341(79.67)		
Whether there is intestinal obstruction					
Yes	6	2(3.70)	4(0.94)	2.991	0.084
No	476	52(96.30)	424(99.06)		
Previous frequency of rectal resection(times)		1.43 ± 0.37	1.34 ± 0.35	(1.769)	0.078

NRS2002 refers to the Nutritional Risk Screening 2002

### LASSO-Logistic regression analysis of risk factors for anastomotic leakage after low rectal cancer surgery

**Fig. 2:**
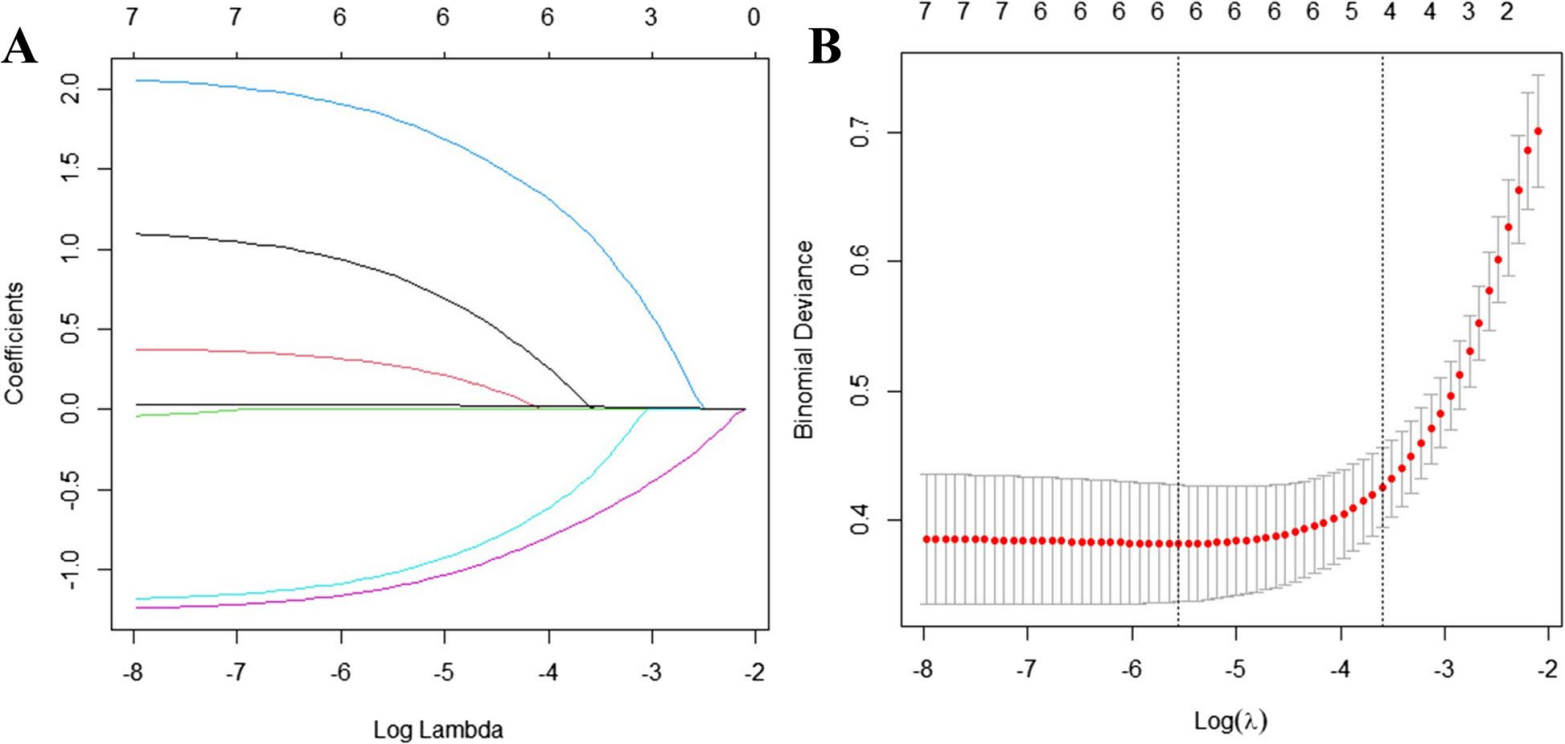
LASSO-Logistic regression analysis **A**: LASSO regression coefficient plot; **B**: 10-fold cross-validation plot for LASSO regression dimensionality reduction

Using postoperative anastomotic leakage (AL) occurrence in the training cohort as the dependent variable (“AL group” coded as 1, “N-AL group” coded as 0), and the variables with significant differences in Table 1 — gender (“male” coded as 1, “female” coded as 0), maximum tumor diameter (“≥5 cm” coded as 1, “<5 cm” coded as 0), tumor T stage (“≥T3” coded as 1, “≤T2” coded as 0), NRS2002 score (“≥3” coded as 1, “<3” coded as 0), protective stoma (“yes” coded as 1, “no” coded as 0), tumor distance from the anal verge (continuous variable), and operative time (continuous variable) — as independent variables (a total of 7 variables), LASSO regression analysis was performed using R software version 4.5.0.The results indicated that when the penalty parameter λ = 0.02735463, the model showed optimal performance, and five influencing factors were ultimately selected: gender, NRS2002 score, protective stoma, tumor distance from the anal verge, and operative time (see Figs. 2).Collinearity diagnostics for these five factors showed variance inflation factors (VIFs) all less than 10, indicating no collinearity or interaction among the variables.These five non-collinear factors were further included in logistic regression analysis. The results demonstrated that gender (OR = 3.107), NRS2002 score (OR = 8.619), protective stoma (OR = 0.297), tumor distance from the anal verge (OR = 0.284), and operative time (OR = 1.033) were all significant influencing factors for postoperative AL in low rectal cancer patients (*P* < 0.05). For details, see Table 2.

**Table 2:** Logistic regression analysis

Influencing factors	B	SE	Wald	*P*	OR	95%CI
Gender	1.134	0.498	5.192	0.023	3.107	1.172 ~ 8.238
NRS2002 rating	2.154	0.487	19.541	0.000	8.619	3.317 ~ 32.440
Prophylactic stomy	−1.213	0.481	6.374	0.012	0.297	0.116 ~ 0.762
Distance from tumor to anal margin	−1.258	0.204	38.074	0.000	0.284	0.191 ~ 0.424
Operation time	0.032	0.005	36.830	0.000	1.033	1.022 ~ 1.044
Constant	−2.939	1.270	5.358	0.021	0.953	-

### Nomogram model

**Fig. 3:**
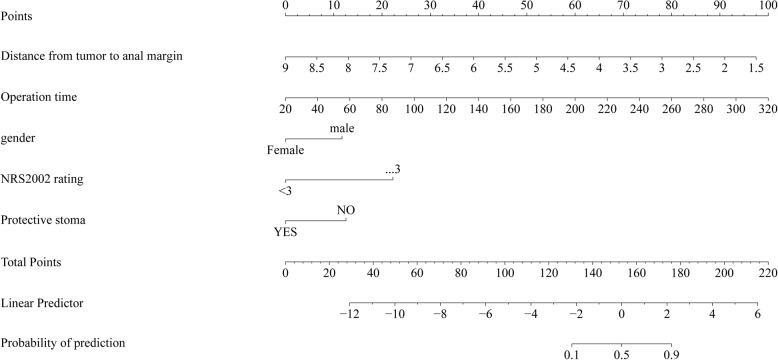
Nomogram model

The factors identified in Table 2—gender, NRS2002 score, prophylactic ostomy, distance of the tumor from the anal verge, and operation time—were used to construct a nomogram model for predicting AL using R software. The resulting nomogram is shown in Fig. 3. Score each factor according to its corresponding scale above. The total score is the sum of all individual factor scores. This total score corresponds to the scale below and is used to predict the risk of anastomotic leakage (AL) after surgery for low rectal cancer.For example: Patient A is male (score: 12.5), has an NRS 2022 score ≥ 3 (score: 24.0), did not undergo a protective stoma (score: 13.0), had an operation time of 180 min (score: 53.0), and the tumor was located 5 cm from the anal verge (score: 52.0). The total score is 154.5, which corresponds to an approximate 52% risk of developing AL.

### Validation of nomogram model

**Fig. 4:**
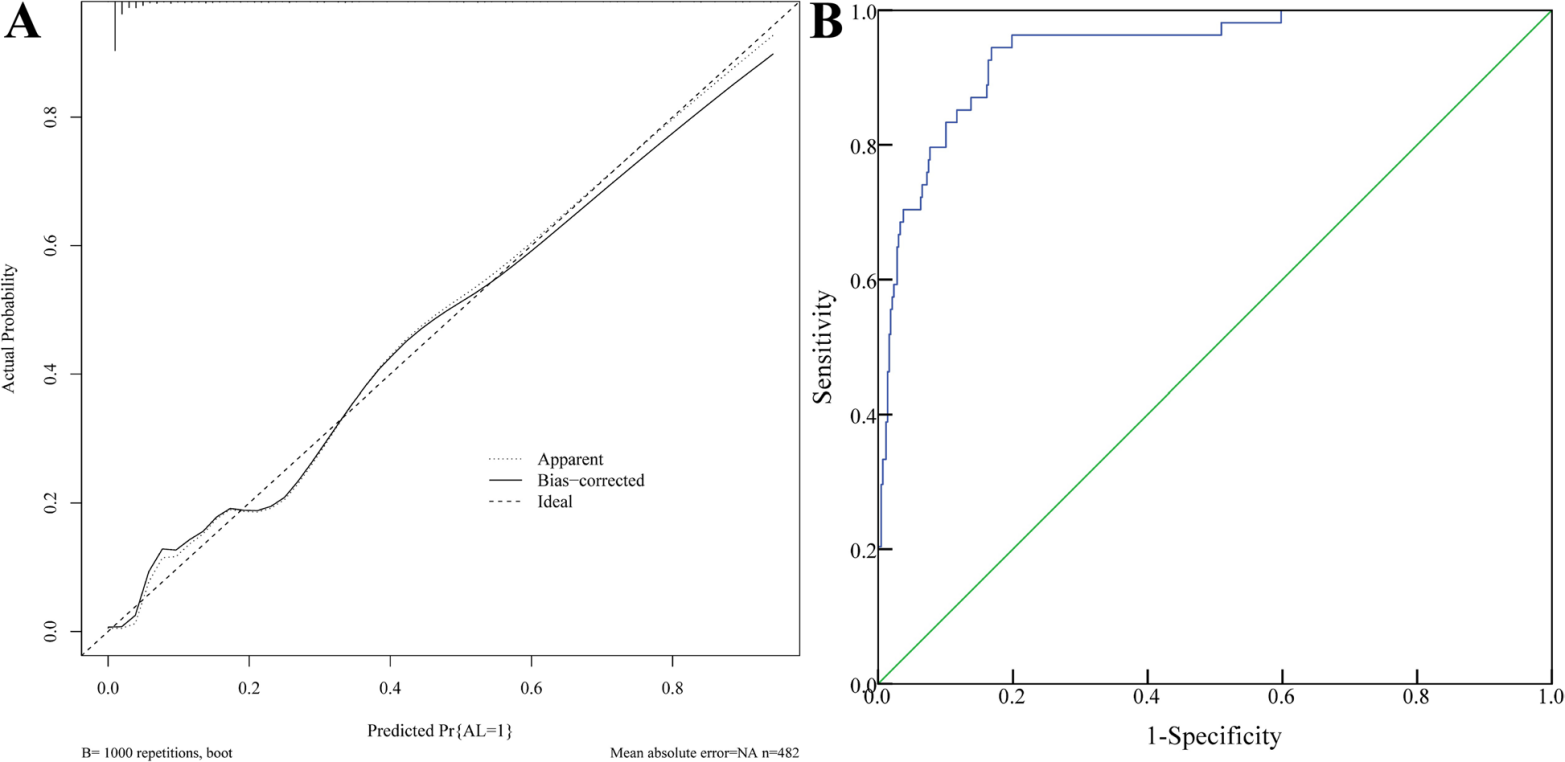
Validation of Nomogram Model **A**: Calibration curve for the training set; **B**: ROC curve for the training set

The Hosmer-Lemeshow goodness-of-fit test was used to evaluate model calibration.The results showed that there was no statistically significant difference between the model’s predicted values and the actual observed values (χ²=6.438, *P* = 0.598). In addition, bootstrap validation with 1,000 resamples showed that the slope of the calibration curve approached 1, suggesting good consistency.The calibration curve slope approached 1, demonstrating good calibration. Model discrimination was assessed using the receiver operating characteristic (ROC) curve, which yielded an area under the curve (AUC) of 0.940 (95% CI: 0.915–0.960), indicating good discrimination. The Brier score was used to evaluate the accuracy of the predictive model, with a Brier score of 0.0518, indicating good accuracy. See Fig. 4.

### Comparison of influencing factors between the AL and N-AL groups in the validation set

General Clinical Data of Patients in the Training and Validation Sets: There were no significant differences between the training and validation sets in terms of gender (χ² = 3.127, *P* = 0.077), NRS2002 score (χ² = 0.038, *P* = 0.845), protective stoma (χ² = 0.291, *P* = 0.590), tumor distance from the anal verge (t = 0.854, *P* = 0.394), or operation time (t = 0.733, *P* = 0.464), with all *P*-values > 0.05.Among the 127 patients in the validation set, 16 patients developed postoperative AL (AL group), while 111 did not (N-AL group). In the validation set, the AL group had a higher proportion of male patients, a higher proportion of patients with NRS2002 scores ≥ 3, and longer operative times compared to the N-AL group. Conversely, fewer AL patients in the validation set had a protective stoma, and the tumor was located a shorter distance from the anal verge than in the N-AL group (*P* < 0.05).See Table 3 for details.

**Table 3:** Comparison of influencing factors between the two patient groups in the validation set

Factor		AL group(*n* = 16)	N-AL group(*n* = 111)	χ^2^(t)	*P*
Gender					
Male	67	13(81.25)	54(48.65)	5.963	0.015
Female	60	3(18.75)	57(51.35)		
NRS2002 rating					
< 3	70	4(25.00)	66(59.46)	6.713	0.010
≥ 3	57	12(75.00)	45(40.54)		
Prophylactic stomy					
Yes	107	10(62.50)	97(87.39)	6.528	0.011
No	20	6(37.50)	14(12.61)		
Distance from tumor to anal margin(cm)	-	3.85 ± 0.76	5.11 ± 1.04	(4.663)	< 0.001
Operation time(min)	-	184.26 ± 40.17	142.15 ± 36.41	(4.270)	< 0.001

### Validation of the nomogram model using the validation set

**Fig. 5:**
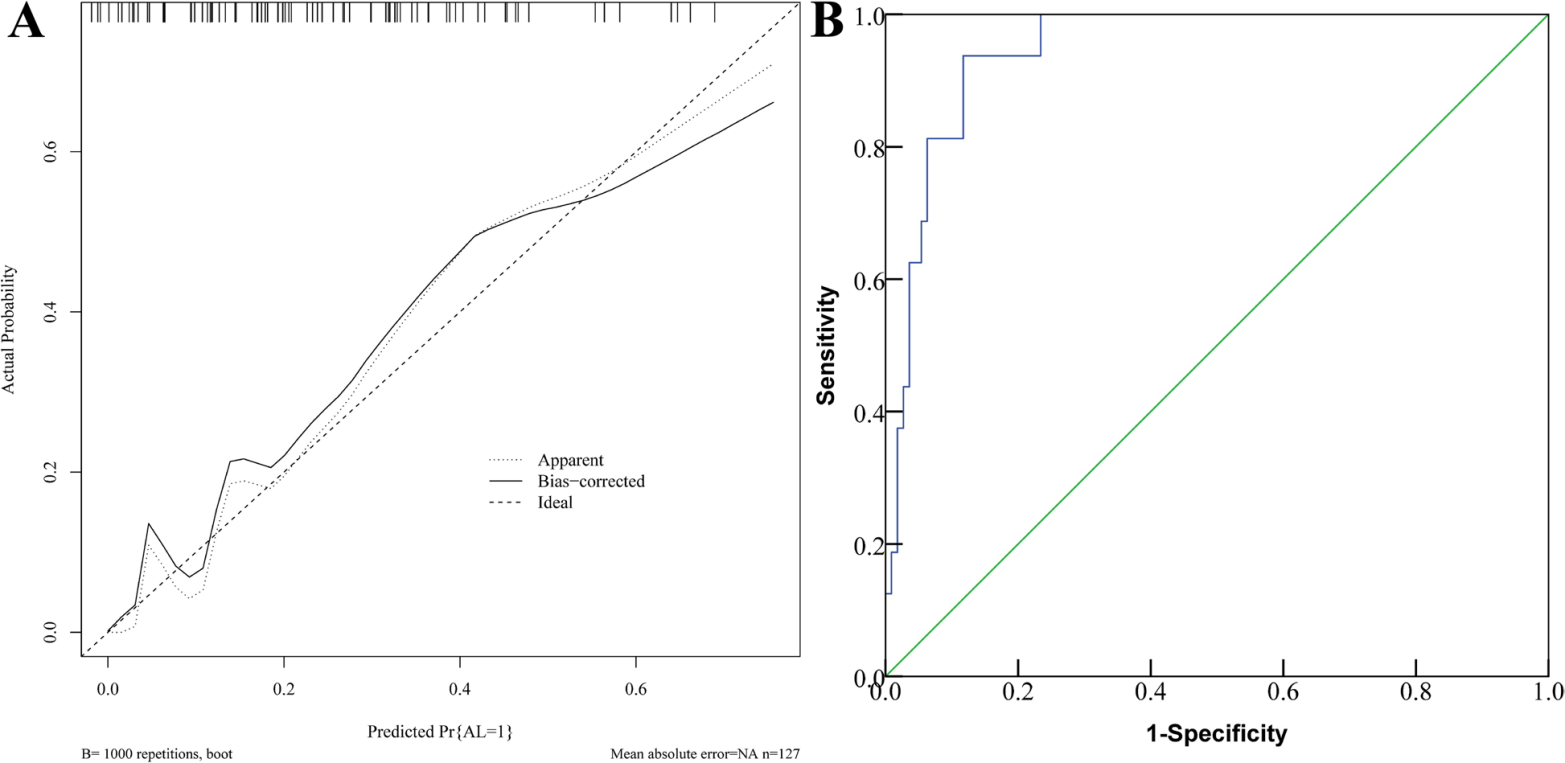
Validation of the Nomogram Model Using the Validation Set **A**: Calibration curve for the validation set; **B**: ROC curve for the validation set

The nomogram model was validated using the five influencing factors from patients in the validation set. The results showed that the Hosmer-Lemeshow (H-L) goodness-of-fit test indicated no statistically significant difference between the predicted and actual values (χ² = 2.662, *P* = 0.954). The calibration curve had a slope close to 1, and the area under the ROC curve (AUC) was 0.947 (95% CI: 0.893–0.979), and the Brier score was 0.0613,demonstrating good model calibration, discrimination and accuracy.See Figs. 5.

### Clinical application value of the nomogram model

**Fig. 6:**
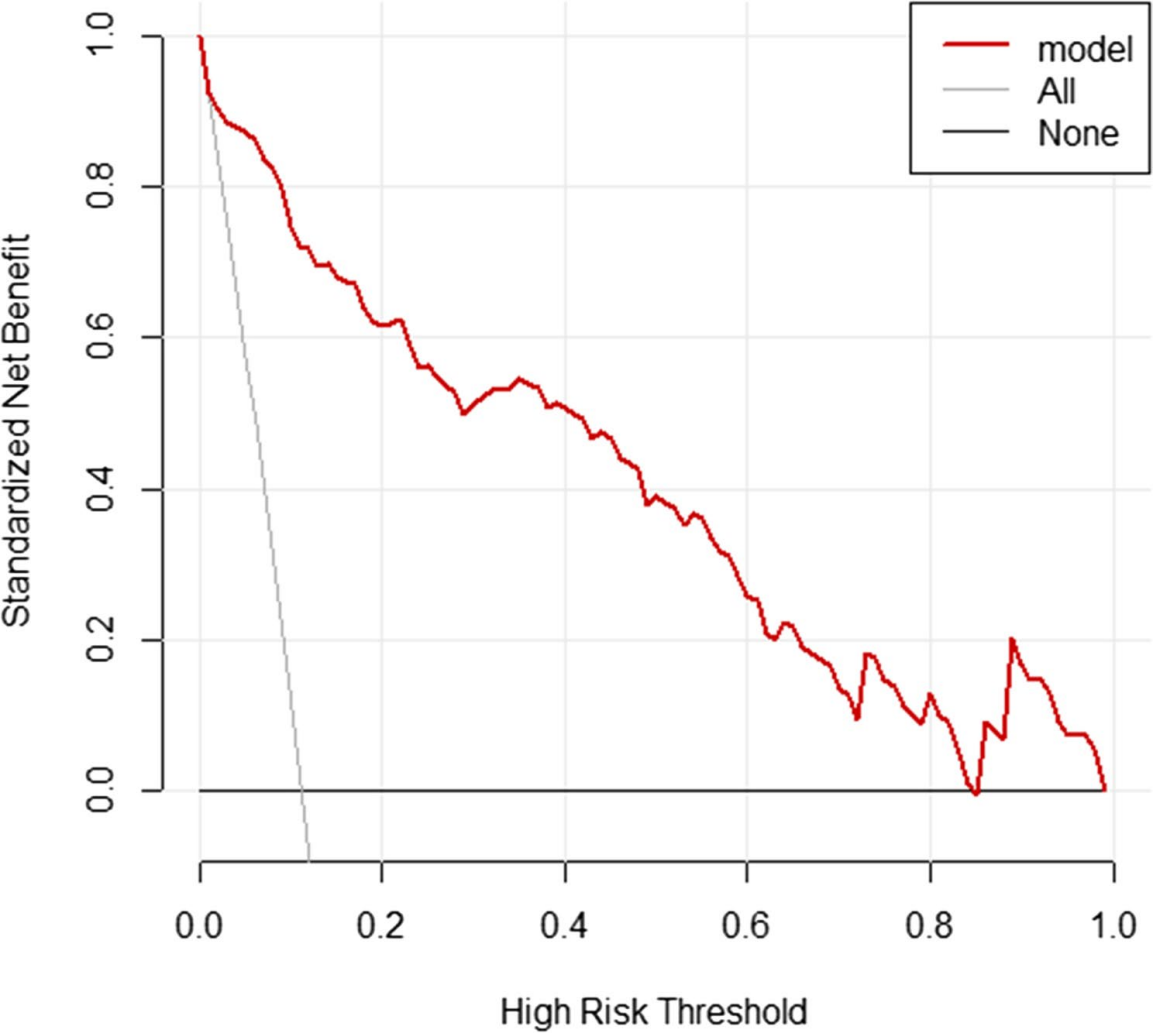
DCA curve

A Decision Curve Analysis (DCA) curve was plotted to evaluate the clinical utility of the nomogram model in predicting the risk of postoperative anastomotic leakage (AL) in low rectal cancer patients. According to the DCA curve, when the predicted risk threshold ranges from 0.08 to 0.85, tthe model demonstrates high clinical utility and net benefit.See Fig. 6.

## Discussion

Due to individual differences, regional variations, hospital equipment, surgical parameters, intraoperative blood loss, and surgical techniques, the risk of postoperative anastomotic leakage (AL) in low rectal cancer patients also varies [11, 12]. Predicting the risk of AL based on different influencing factors has positive implications for improving patients’ postoperative quality of life.

In this study, 482 patients with rectal cancer were enrolled in the training set from June 2017 to May 2023, and 127 patients were included in the validation set from June 2023 to April 2025. All patients in both sets had low rectal cancer located within 8 cm from the anal verge and were treated at the same hospital, minimizing the impact of surgeon, surgical technique, and regional differences on the study results. Among the 482 patients in the training set, 54 developed postoperative AL, while 428 did not, resulting in an AL incidence rate of 11.20%, which is consistent with previous studies [3].

This study compared the general clinical data between the AL and N-AL groups in the training set and found that the AL group had a higher proportion of male patients, patients with tumors with a maximum diameter ≥ 5 cm, patients with tumor stage ≥ T3, and patients with an NRS2002 score ≥ 3, and experienced longer operative times compared to the N-AL group. In contrast, in the AL group, the tumor was located a shorter distance from the anal verge, and a lower proportion of patients had a protective stoma (*P* < 0.05).Further analysis using Lasso-logistic regression identified five factors—gender, NRS2002 score, protective stoma, tumor distance from the anal verge, and operation time—as independent risk factors for postoperative AL. Based on these five factors, a predictive model was constructed with the following results:

Male gender (OR = 3.107) was identified as a risk factor for postoperative anastomotic leakage (AL) in patients with low rectal cancer. In this study, the proportion of male patients was relatively high, which aligns with the commonly recognized trend that rectal cancer is more prevalent in males. Moreover, the incidence of postoperative AL was higher in male patients than in female patients. According to the nomogram model, being male adds 12.5 points to the total risk score.The possible reasons for this may include: the male pelvis is narrower than the female pelvis, providing a smaller operative space. This makes rectal transection and anastomosis more challenging in males, increasing the likelihood of extended operation time, greater blood loss, and deviations in intraoperative cutting angles. These factors may lead to repeated cuts, collectively increasing the risk of postoperative AL. Thus, male rectal cancer patients face a higher risk of AL. Penna et al. [13] conducted a prospective study across 29 countries, involving 107 surgical centers, where 1,594 consecutive reconstructive resection cases were recorded over 30 months to determine the incidence of anastomosis-related complications after transanal total mesorectal excision. The study identified four high-risk factors for anastomotic failure, including male gender, consistent with the findings of this research. This suggests that surgeons should pay extra attention to male patients, aiming to minimize operation time, blood loss, and other factors that might increase AL risk during the procedure. By reducing the incidence of AL, surgeons can improve postoperative quality of life for patients.

An NRS2002 score ≥ 3 (OR = 8.619) was identified as a significant risk factor for postoperative anastomotic leakage (AL) in patients with low rectal cancer. The nomogram model further indicates that when the NRS2002 score is ≥ 3, it contributes an additional 24.0 points to the total risk score. In China, hospitalized patients’ nutritional status is typically assessed using the NRS2002 system, a highly reliable tool for evaluating nutritional status. Previous studies have demonstrated the value of NRS2002 in assessing postoperative nutritional levels in cancer patients. For example, Tian et al. [14]found that NRS2002 is suitable for preoperative nutritional screening in cervical cancer patients. Lee et al. [15] evaluated the relationship between postoperative NRS scores and AL in rectal cancer surgeries, finding that the NRS score is an independent predictor of postoperative AL. Similarly, Zhang et al. [16], in their analysis of the relationship between nutritional status and surgical prognosis, concluded that an NRS2002 score of 5 or above is a risk factor for poor postoperative outcomes in gastrointestinal tumors. These studies indicate that nutritional risk is a significant factor for adverse postoperative outcomes. This is because patients with poor preoperative nutritional status lack sufficient physical function and essential reserves, resulting in inadequate nutritional support during the postoperative recovery period to meet the body’s needs. Consequently, poor anastomotic healing and slower healing rates increase the risk of AL. Therefore, patients with an NRS2002 score of 3 or above, indicating higher nutritional risk, are more likely to develop postoperative AL. These findings suggest that physicians should conduct targeted nutritional risk screening for rectal cancer patients before surgery. For patients with significant nutritional risks, appropriate nutritional support should be provided, and surgery should be scheduled at an optimal time to ensure sufficient nutritional reserves during and after the procedure to mitigate the damage caused by surgery, promote wound healing, and reduce the risk of AL.

protective stoma (OR = 0.297) was identified as a protective factor influencing the occurrence of postoperative anastomotic leakage (AL). The absence of a protective stoma increases the nomogram model score by 13.0 points. Currently, it is widely agreed (both in domestic and international studies) that establishing a protective stoma is an important measure for reducing AL rates. This procedure can also reduce the mortality and recurrence rates associated with AL. However, some patients experience fear or resistance toward stoma creation, or have aversion to undergoing a second surgery, leading them to refuse a protective stoma, which increases the risk of postoperative anastomotic leakage.Veenhof et al. [17] conducted a randomized multicenter trial involving 234 patients, demonstrating that diverting the anastomosis reduces the occurrence of symptomatic AL after rectal cancer surgery. Shimizu et al. [18] studied 226 rectal cancer patients who underwent laparoscopic surgery and found that ileostomy can reduce the risk of AL in malnourished male patients. These findings align with the results of this study. However, there is still a lack of meta-analyses on the necessity of protective stoma for low rectal cancer patients. Therefore, this predictive model can be used in clinical practice to help persuade patients to undergo protective stoma, which is of great significance in reducing the incidence of anastomotic leakage (AL).

Tumor distance from the anal verge (OR = 0.284) was also identified as a risk factor for postoperative anastomotic leakage (AL). A shorter distance is associated with a higher risk of AL. For every 0.5 cm decrease in tumor distance from the anal verge, the nomogram model score increases by 7.3 points. Fukada et al. [19] investigated AL risk factors in 101 rectal cancer patients and found that tumors located less than 6 cm from the anal verge were significantly associated with AL. Zheng et al. [20]conducted a retrospective analysis of 2,618 rectal cancer patients to identify factors predicting postoperative AL risk, concluding that the tumor-to-anal verge distance is a critical determinant of AL risk. These findings are consistent with the results of this study. The increased risk can be attributed to the greater difficulty of performing anastomosis as the tumor location becomes lower. Lower tumor locations require more intestinal mobilization to achieve tension-free anastomosis, while the intraluminal pressure increases as the anastomosis site moves lower, further elevating the risk of postoperative AL.

Longer operation time (OR = 1.033) was identified as a factor associated with an increased risk of postoperative anastomotic leakage (AL). According to the nomogram model, for every 20-minute increase in operation time, the total score increases by 7.5 points.Extended operative time may indicate more difficult and complex surgical procedures. High-complexity surgeries can lead to greater surgical trauma, thereby increasing the likelihood of AL in postoperative patients. The Italian Colorectal Cancer Collaborative Group analyzed data from 24 referral centers in Italy to identify risk factors for anastomotic leakage after rectal cancer anterior resection. Their analysis revealed a close association between longer operation times and higher AL risk [21]. These findings suggest that in clinical practice, surgeons should continuously improve their skills and operational proficiency to reduce operative time whenever possible. For patients undergoing lengthy surgeries, targeted monitoring of anastomotic healing is essential to prevent postoperative AL.

Application example of the nomogram model: For instance, Patient A is male, has an NRS2002 score ≥ 3, did not undergo a protective stoma, had an operation time of 180 min, and the tumor was located 5 cm from the anal verge. These factors correspond to the following scores in the nomogram model: 12.5 points for male gender, 24.0 points for NRS2002 score ≥ 3, 13.0 points for no protective stoma, 53.0 points for operation time, and 52.0 points for tumor distance. The total score is 154.5, which corresponds to an estimated AL risk of approximately 52%.This suggests a moderate risk of postoperative AL for this patient. To reduce this risk, preoperative nutritional support can be provided, a protective stoma may be considered during surgery, and efforts should be made to minimize operation time. Postoperatively, close monitoring of the patient’s physical condition and ensuring adequate nutritional supply can help improve prognosis and quality of life. The above practical application suggests that, in clinical decision-making, efforts should be made to shorten the operation time, to persuade patients to undergo protective stoma when their physical condition permits, and to actively provide nutritional support to reduce the NRS2022 score. Additionally, for male patients and those with tumors located closer to the anal verge, surgical procedures should be performed with greater precision to avoid adverse factors such as increased intraoperative bleeding or deviations in the angle of dissection. The nomogram model was subsequently validated and shown to have good practicality. There was no statistically significant difference between the model’s predicted values and the actual observed values (χ² = 6.438, *P* = 0.598), indicating no overfitting. The slope of the calibration curve approached 1, suggesting good consistency. The AUC was 0.940 (95% CI: 0.915–0.960), indicating good discrimination. The Brier score was 0.0518, reflecting good accuracy. Overall, the model performed excellently, which may be attributed to the training dataset being accurate, complete, and highly representative. However, relying solely on the training set for validation may limit reliability. Therefore, an additional 127 patients collected between June 2023 and April 2025 were included in a validation set. The validation dataset is independent and represents the target population for the model’s intended future application, demonstrating its external validation capability. The validation results also indicate that the model has a certain level of practical applicability. The model performed well on both the training and validation datasets, suggesting a low risk of overfitting. Furthermore, Decision Curve Analysis (DCA) indicated that when the predicted risk threshold for postoperative AL ranges from 0.08 to 0.85, the nomogram model offers high clinical utility and net benefit, demonstrating good clinical utility. Thus, it is important to monitor the above-mentioned factors in rectal cancer patients. For those at high risk of AL, it is essential to develop personalized surgical plans to further reduce the risk of this complication.

In conclusion, gender, NRS2002 score, prophylactic ostomy, tumor distance from the anal verge, and operation time are significant factors influencing postoperative AL in low rectal cancer patients. Based on these factors, the nomogram model developed in this study demonstrated good consistency, discriminative ability, and clinical applicability. Due to the unique characteristics of patients undergoing emergency surgery, those requiring multiorgan resection, or those with distant metastases, such high-risk individuals were excluded from this study to ensure a more homogeneous patient population. Therefore, the nomogram model is not applicable to these excluded cases. In clinical practice, it is important to recognize that this model is intended for use in patients with low rectal cancer who are undergoing elective surgery and have no distant metastasis.Additionally, this study was conducted at a single center, with a relatively small validation sample size, all sourced from the same hospital. As a result, factors such as physician experience, surgical techniques, and intraoperative procedures were relatively consistent, and the model may not capture variability across different regions. This could limit the generalizability of the findings. In future research, multicenter studies with larger sample sizes from various regions will be conducted to further validate the clinical utility of this nomogram model and enhance its generalizability.

## Supplementary Information

Supplementary Material 1.

## Data Availability

The datasets used during the present study are available from the corresponding author upon reasonable request.
