# Supplementary material for: Building a risk prediction model for anastomotic leakage postoperative low rectal cancer based on Lasso-Logistic regression
Source: BMC Gastroenterol. 2025 Jul 30;25:540. doi: 10.1186/s12876-025-04128-y (PMC12309234; doi:10.1186/s12876-025-04128-y)

To whom it may concern:

This memo is to certify that the paper titled *s Building a risk prediction model for anastomotic leakage postoperative low rectal cancer based on Lasso-Logistic regression* has been edited for language by ONCE-TRANSLATION, a company dedicated to helping international researchers publish their findings in the best English language journals possible.

Our international paper editing service is performed by a subject expert editor and approved by two senior editors.

The certificate is being issued upon the request of the client. If you have any questions, please contact info@once-translation.com

Seal of company:

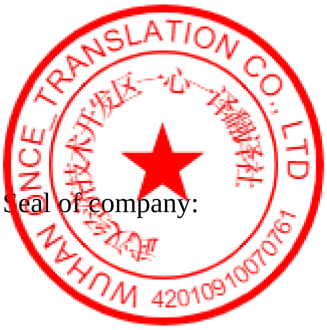

Supplement: Supplementary file 1 — Supplementary Material 1. [file 12876_2025_4128_MOESM1_ESM.pdf]
